# Evaluating an urgent care antibiotic stewardship intervention: a multi-network collaborative effort

**DOI:** 10.1017/ice.2024.213

**Published:** 2025-03

**Authors:** Daniel E. Park, Annie L.S. Roberts, Rana F. Hamdy, Sabrina Balthrop, Patrick Dolan, Cindy M. Liu

**Affiliations:** 1 Department of Environmental and Occupational Health, George Washington University, Washington, DC, USA; 2 Children’s National Hospital, Washington, DC, USA; 3 Department of Pediatrics, George Washington University School of Medicine and Health Sciences, Washington, DC, USA; 4 Urgent Care Association, Batavia, IL, USA; 5 PM Pediatrics, Mount Prospect, IL, USA

## Abstract

**Objective:**

Urgent care centers (UCCs) have reported high rates of antibiotic prescribing for acute respiratory tract infections. Prior UCC studies have generally been limited to single networks. Broadly generalizable stewardship efforts targeting common diagnoses are needed. This study examines the effectiveness of an antibiotic stewardship intervention in reducing inappropriate prescribing for bronchitis and viral upper respiratory tract infections (URTIs) in UCCs.

**Design:**

A quality improvement study comparing inappropriate antibiotic prescribing rates in UCCs after the introduction of an antibiotic stewardship intervention.

**Setting:**

Forty-nine UCCs in 27 different networks from 18 states, including 1 telemedicine site.

**Participants:**

Urgent care clinicians from a national collaborative of UCCs, all members of the Urgent Care Association.

**Methods:**

The intervention included signing a commitment statement and selecting from 5 different intervention options during 3 plan-do-study-act cycles. The primary outcome was the percentage of urgent care encounters for viral URTIs or bronchitis with inappropriate prescribing, stratified by clinician engagement and diagnosis. A 3-month baseline and 9-month intervention period were compared using a regression model using a generalized estimating equation.

**Results:**

Among 15,385 encounters, the intervention was associated with decreases in inappropriate antibiotic prescribing for bronchitis (48% relative decrease, aOR = 0.52; 95% CI, 0.33–0.83) and viral URTIs (33%, aOR = 0.67; 95% CI, 0.55–0.82) among actively engaged clinicians compared to baseline. The intervention did not result in significant changes for clinicians not actively engaged.

**Conclusions:**

This intervention was associated with reductions in inappropriate prescribing among actively engaged clinicians. Implementing stewardship interventions in UCCs may reduce inappropriate antibiotic prescriptions for common diagnoses; however, active clinician engagement may be necessary.

## Introduction

Inappropriate antibiotic prescribing is a main driver of antimicrobial resistance. Most antibiotic prescribing occurs in outpatient settings, where approximately 30% of outpatient antibiotic prescriptions are inappropriate.^1,2^ Urgent care centers (UCCs) have been reported among the outpatient settings to have high rates of inappropriate prescribing. As one of the fastest-growing outpatient settings, UCCs have a responsibility to implement generalizable stewardship interventions to counter high rates of inappropriate antibiotic prescribing.^3–6^ Approximately 44% of outpatient antibiotic prescriptions are for acute respiratory conditions.^7^ Bronchitis and viral upper respiratory tract infections (URTIs) together make up 24% of these acute respiratory conditions, accounting for approximately 10.5% of outpatient antibiotic prescriptions.^7^ These conditions are important targets for reducing inappropriate prescribing, especially in outpatient settings.

Outpatient antibiotic stewardship interventions have been associated with decreased antibiotic prescribing.^8^ A recent study showed that implementation of an antibiotic stewardship intervention within a single urgent care network was associated with reduced rates of inappropriate prescribing for respiratory conditions.^9^ Most studies that evaluate antibiotic stewardship in adult populations are conducted in a single network or include a small number of sites.^9–12^ Other cross-network interventions have either not primarily focused on UCCs^13,14^ or are focused largely in the pediatric space.^15–17^ One study implemented across 20 pediatric UCCs observed a 32.5% reduction in inappropriate prescribing for otitis media and pharyngitis, which are diagnoses often targeted by pediatric-focused antibiotic stewardship efforts.^18,19^

This study seeks to understand the effect of broadly generalizable antibiotic stewardship interventions on a large, geographically diverse set of UCCs for common diagnoses of bronchitis and viral URTIs, across patients of all ages.

## Methods

### Ethics

The George Washington University IRB waived informed consent for this quality improvement study (NCR224504). The SQUIRE 2.0 reporting guidelines were followed (Supplemental Table 1).

**Table 1.** Urgent care center encounter patient characteristics

Characteristic	Baseline period	Intervention period	Overall
Total visits	4,439	10,946	15,385
**Race/ethnicity**			
Black	524 (11.8%)	1050 (9.6%)	1,574 (10.2%)
White	2,726 (61.4%)	7,090 (64.8%)	9,816 (63.8%)
Hispanic	335 (7.6%)	969 (8.9%)	1,304 (8.5%)
Other	854 (19.2%)	1,837 (16.8%)	2,691 (17.5%)
**Insurance**			
Commercial	2,765 (62.3%)	6,749 (61.7%)	9,514 (61.8%)
Public	1,236 (27.8%)	3,223 (29.5%)	4,459 (29.0%)
Military	57 (1.3%)	161 (1.5%)	218 (1.4%)
None	158 (3.6%)	356 (3.3%)	514 (3.3%)
Unsure	223 (5.0%)	456 (4.2%)	679 (4.4%)
Missing	0	1	1
**Age**			
0–11 m	101 (2.3%)	286 (2.6%)	387 (2.5%)
1–20 y	2,160 (48.7%)	4,965 (45.4%)	7,125 (46.3%)
21–40 y	1,027 (23.1%)	2,756 (25.2%)	3,783 (24.6%)
41–60 y	678 (15.3%)	1,671 (15.3%)	2,349 (15.3%)
61–80 y	424 (9.6%)	1,138 (10.4%)	1,562 (10.2%)
81 and over	49 (1.1%)	130 (1.2%)	179 (1.2%)

### Study setting

Participating UCCs included UCCs with membership in the Urgent Care Association (UCA). UCC clinician participation was coordinated by the UCA, a trade association for urgent care.

### Study population

Clinicians (MDs, DOs, or APPs) from each site randomly selected 30 charts to submit per month per UCC with a primary diagnosis of either bronchitis or viral URTI, identified by ICD-10 codes (Supplemental Table 2), and entered de-identified data into a REDCap database via direct entry surveys or through data uploads.^20^ The number of charts was standardized across clinics and was selected for clinician feasibility, especially at clinics without resources to automate data extraction. The random selection included both diagnoses to reflect a representative distribution of bronchitis and viral URTI (including COVID-19) encounters. Clinicians reviewed charts chosen at random from the entire UCC where they worked, and not solely charts from their own patients. Encounters of all ages with a bronchitis or viral URTI diagnosis were included. Encounters were excluded from the analysis if they had concurrent primary or secondary diagnoses that could warrant an antibiotic (Supplemental Table 3).^15^

De-identified data collected included demographic information, diagnosis, whether or not an antibiotic was prescribed, prescription type (prescription to be filled immediately, to be picked up after the visit, or picked up if symptoms worsened after the visit), what antibiotic was prescribed, the duration and frequency of the prescription, and concurrent diagnoses, along with clinician engagement status. The research team provided daily chart tracking numbers, and tools such as a comprehensive data dictionary were used to facilitate correct chart extraction; we did not do a manual review of charts to assess misdiagnoses.

### Study design

The UCA and Urgent Care Foundation recruited participants by sending out invitations via email and newsletters, inviting clinicians and centers to participate. Participation was determined at the clinician level, and all participating centers had at least 1 active clinician. The 12-month study included 3 months of baseline (pre-intervention) data collection (September 2021 to November 2021) and 9 months of intervention (December 2021 through August 2022).

The antibiotic stewardship intervention consisted of 3 plan-do-study-act (PDSA) cycles. During the first PDSA cycle (end of baseline), participating clinicians signed the UCA/College of Urgent Care Medicine Antibiotic Stewardship Commitment Statement and chose intervention(s) to implement from the available package of validated materials.^21^ The second and third PDSA cycles (in months 3 and 5 of the intervention) allowed clinicians to review progress and select new intervention(s) to implement if desired. These interventions included patient education handouts, patient engagement materials (videos, articles, letter templates, etc.), clinician education, treatment guidelines, and signage/social media materials. The package of materials is publicly available and described in Supplemental Table 4.^21^ Each site was able to select the specific materials they thought would be most effective for their given UCC setting. We did not track usage or evaluate the efficacy of individual materials.

Clinicians committed to active participation in data collection, implementing stewardship efforts, attending a minimum of 4 webinars, and regular monthly feedback for the entire study period. Active engagement was based on self-reported involvement in the intervention. Clinicians working at participating centers but not actively involved in the above activities were considered to be not actively engaged. As some interventions were aimed to be broadly applicable to the center, regardless of engagement in study activities, clinicians who were not actively engaged were eligible to have their charts randomly selected and extracted for the study. The subject matter of the webinars included a review of overall study prescribing patterns, cross-site learnings, highlight of a specific intervention, quality improvement education, clinical best practices for common conditions, data updates, and a questions and answers session. Site-specific inappropriate antibiotic prescribing rates were also provided to each actively engaged clinician, monthly. Clinicians noted during the chart extraction whether the selected patient encounter involved a clinician actively engaged in the study or a clinician not actively engaged in the quality improvement study.

As an incentive for active participation, clinicians were offered Type 2 Maintenance of Certification (MOC) credits from the American Board of Internal Medicine and the American Board of Pediatrics and Type 4 MOC from the American Board of Medical Specialties. These credits coincided with the submission of the final project evaluation. Active engagement was defined as attendance at a minimum of 4 of 9 monthly webinars during the 9-month intervention period, active participation in the implementation of the selected interventions, and participation in feedback and evaluation mechanisms. Feedback included monthly data reports on site-level prescribing rates and overall study prescribing rates. Webinars were held monthly to review data collection progress, aggregate results, and discuss successes and challenges.

### Intervention outcomes

Information was collected on how the antibiotic was prescribed to the patient: a prescription to be filled immediately at the UCC, to be picked up by the patient after the visit, or to be picked up if symptoms worsened after the visit. Antibiotic prescribing was defined as inappropriate if the clinician indicated yes to any of these options. The primary outcome measure for this study was inappropriate antibiotic prescribing for bronchitis and viral URTI diagnoses overall and also stratified by individual diagnosis and clinician active engagement status. Basic demographic information was also extracted from the patient chart. The primary study outcome was the change in inappropriate prescribing for bronchitis or viral URTI diagnoses, comparing baseline (September to November 2022) and intervention periods (December 2022 to August 2023). The SMART aim was to achieve a 20% relative decrease in inappropriate antibiotic prescribing from baseline to 9 months after the intervention was initiated.

### Data analysis

Data were analyzed using SAS version 9.4. Demographic characteristics for the encounters were compared using a χ^2^ test. χ^2^ tests were used to compare proportions of antibiotic prescribing across the 2 study periods, including comparisons from baseline to the final 2 months of the intervention. Comparisons were made for antibiotic prescribing overall, and also by primary diagnosis. Statistical process control (SPC) charts were developed to assess how antibiotic prescribing changed over the 9-month intervention period (Supplemental Figure 1). Along with monthly reports generated to assess overall progress, monthly individualized site reports were shared with the sites and indicated trends in inappropriate antibiotic prescribing (including through SPC charts) and whether those changes were statistically significant.

A binomial generalized estimating equation model was used to evaluate the association between the antibiotic stewardship intervention and inappropriate prescribing overall and for both diagnoses, with clustering by each UCC to address within-UCC prescribing variability. Odds ratios were calculated to assess the difference in antibiotic prescribing during the intervention period compared to the baseline period, adjusted *a priori* for age and race/ethnicity. For secondary analyses, the model was stratified by primary diagnosis and by clinician participation status. Both models used an exchangeable correlation structure.

## Results

Forty-nine UCCs from 18 different states participated. These sites represented 27 different urgent care networks. A total of 138 clinicians were actively engaged in the study, with each site providing up to 4 clinicians (Supplemental Figure 2). Among the 15,385 patient encounters, 2,482 resulted in 1 or more antibiotic prescriptions (16.0%), and 170 encounters were excluded. The mean age of the patients included in the collected encounters was 26.3 (SD, 23.0) years. Demographic information of these encounters is presented in Table 1. Of all encounters, 49.2% involved a clinician actively engaged in the study, and 50.7% involved clinicians not actively engaged (Supplemental Table 5).

### Antibiotic prescribing before and after intervention

In the baseline period, there were 4,439 encounters, with 3,866 viral URTI encounters and 573 bronchitis encounters. Overall, antibiotic prescribing decreased from 18.0% during baseline to 13.6% by the final 2 months of the intervention (4.4% absolute reduction, 24.4% relative reduction, *P* = < 0.001). However, when accounting for clustering by clinic, inappropriate antibiotic prescribing during the intervention period was not significantly different compared with baseline (aOR = 0.86; *P* = 0.099) (Table 2).

**Table 2.** Antibiotic prescribing by diagnosis during the baseline and intervention periods, stratified by whether the clinician was actively engaged in the quality improvement project

	Inappropriate antibiotic prescribed/number of encounters (%)		Odds of inappropriate prescribing, intervention vs baseline	
	Baseline	Intervention	aOR^a^	*P*-value
**Bronchitis or viral URTI**	799/4439 (18.0)	1683/10946 (15.4)	0.86 (0.72–1.03)	0.099
Actively engaged clinician	333/2100 (15.9)	519/5431 (9.6)	0.61 (0.48–0.77)	**<0.001**
Non-actively engaged clinician	450/2310 (19.5)	1168/5476 (21.3)	1.08 (0.85–1.38)	0.540
**Bronchitis diagnoses overall**	305/573 (53.2)	633/1352 (46.8)	0.63 (0.46–0.87)	**0.005**
Actively engaged clinician	92/230 (40.0)	129/449 (28.7)	0.52 (0.33–0.83)	**0.006**
Non-actively engaged clinician	201/329 (61.1)	502/890 (56.4)	0.66 (0.42–1.01)	0.057
**Viral URTI diagnoses overall**	494/3866 (12.8)	1050/9594 (10.9)	0.92 (0.77–1.10)	0.369
Actively engaged clinician	241/1870 (12.9)	384/4982 (7.7)	0.67 (0.55–0.82)	**<0.001**
Non-actively engaged clinician	249/1981 (12.6)	665/4591 (14.5)	1.17 (0.86–1.58)	0.317

Note. URTI, upper respiratory tract infection.

a Generalized estimating equation, adjusted for age and race/ethnicity, accounting for clustering by clinic.

When stratified by diagnosis, for bronchitis, inappropriate prescribing was 53.2% during baseline and decreased during the intervention period to 38.8% by the final 2 months (14.4% absolute reduction, 27.1% relative reduction, *P* = < 0.001). For viral URTIs, inappropriate prescribing was 12.8% during baseline and decreased during the intervention period to 10.1% by the final 2 months (2.7% absolute reduction, 21.1% relative reduction, *P* = 0.004) (Supplemental Figure 3). Accounting for clustering by clinic, there was a 37% relative reduction for bronchitis (aOR = 0.63; *P* = 0.006) but no significant reduction for viral URTI (aOR = 0.92; *P* = 0.37) (Supplemental Table 6).

### Antibiotic prescribing before and after intervention, by clinician engagement

**Figure 1.**
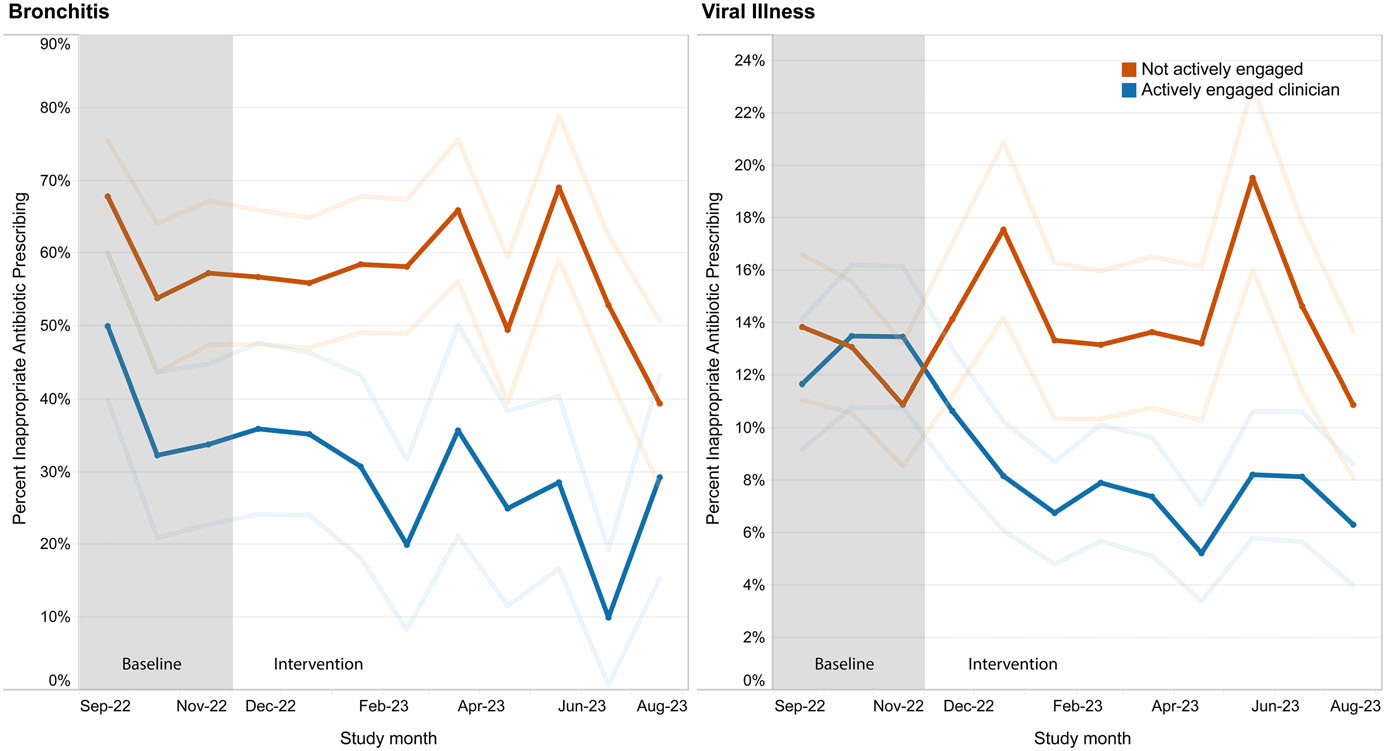
Inappropriate antibiotic prescribing by provider participation in the quality improvement project and by diagnosis. The percentage of urgent care encounters with an inappropriate antibiotic prescription by month, diagnosis, and whether the clinician for the chart was an actively engaged participant in the quality improvement project (blue line) or was not actively engaged in the project (red line). Faded lines represent the 95% confidence intervals for the inappropriate antibiotic prescribing rate. Inappropriate prescribing changes were different between actively engaged clinicians and non-actively engaged clinicians for both bronchitis (*P* < 0.001 for interaction term) and viral upper respiratory tract infections (URTIs) (*P* = 0.036). Prescribing patterns were also different by study engagement among bronchitis diagnoses (*P* = 0.012) but not viral URTIs (*P* = 0.093).

There was a significant decrease in overall inappropriate antibiotic prescribing among clinicians actively engaged in the study, but not among clinicians who were not actively engaged (*P* = 0.002 for interaction by study engagement) (Table 2, Figure 1). Overall, inappropriate prescribing decreased by 39% (aOR = 0.61; 95% CI, 0.48–0.77) in the intervention period compared to baseline for clinicians actively engaged in the quality improvement stewardship intervention, but not for clinicians who were not actively engaged (aOR = 1.08; 95% CI, 0.85–1.38). For bronchitis, antibiotic prescribing significantly decreased by 48% (aOR = 0.52; 95% CI, 0.33–0.83) for clinicians actively engaged in the quality improvement study. Among clinicians not actively engaged, there was a marginally significant decrease in prescribing of 34% (aOR = 0.66; 95% CI, 0.42–1.01). For viral URTIs, antibiotic prescribing decreased by 33% (aOR = 0.67; 95% CI, 0.55–0.82) for clinicians actively engaged in the quality improvement stewardship intervention. Among clinicians not actively engaged in the study, there was no change in inappropriate prescribing for viral URTIs.

### Antibiotic prescribing patterns

During the baseline period, the rate of inappropriate prescribing was highest for White patients (20.2%) compared with Black (11.4%) and Hispanic (14.5%) patients (*P* < 0.001). Inappropriate prescribing rates remained higher among White patients (15.9%) compared with Black (13.1%) and Hispanic (9.9%) (*P* = 0.011) in the intervention period. During the baseline period, prescribing rates for bronchitis diagnoses among clinicians actively engaged in the study were lower compared with clinicians not actively engaged (40.0% vs 61.1%, *P* = 0.012). Baseline prescribing rates for viral URTI diagnoses did not significantly differ between clinicians who were actively and not actively engaged (12.9% vs 12.6%, respectively, *P* = 0.767).

During the intervention period, there was a decrease in the proportion of reviewed charts that involved actively engaged clinicians, with the proportion of clinicians not actively engaged rising from 47.6% to 52.5% between the first 3 and final 3 months (*P* < 0.001). Of all antibiotics given, 17.2% were given during the visit, 76.3% were prescribed to be picked up after the visit, and 6.5% were a watch and wait prescription.

## Discussions

Over 9 months, this project resulted in a substantial reduction of inappropriate prescribing for both bronchitis and viral URTI diagnoses across nearly 50 geographically diverse US UCCs. When stratifying encounters by clinician engagement, reductions in inappropriate prescribing were seen for actively engaged clinicians but not for clinicians who were not actively engaged. For actively engaged clinicians, inappropriate prescribing for bronchitis diagnoses decreased by 48% in the intervention period and decreased by 33% for viral URTI diagnoses.

These results add to the growing evidence on the effectiveness of antibiotic stewardship interventions in UCCs; although many studies have evaluated different endpoints, the reductions in inappropriate prescribing observed in this study are consistent with those in other studies.^9–15^ Notably, the baseline rates of inappropriate antibiotic prescribing were relatively low compared with previously published estimates, particularly for viral URTIs (12.7%); Palms et al. observed urgent care inappropriate prescribing at 75.8% for bronchitis and 41.6% for viral URTIs.^5^ Nonetheless, there was a large and significant reduction (33%) among clinicians actively engaged in the study. The antibiotic stewardship intervention was coordinated through a national trade association of UCCs (UCA) and represented a diverse set of UCCs across 18 states and 27 different networks, suggesting broad generalizability. The primary diagnoses of bronchitis and viral URTIs are commonly occurring diagnoses in people of all ages, also increasing generalizability. Additionally, the stewardship interventions were in line with the Centers for Disease Control and Prevention’s (CDC) Core Elements of Outpatient Antibiotic Stewardship^22^ and similar to multifaceted antibiotic stewardship approaches implemented in other studies.^9,13,18^

Prior studies have suggested that racial differences exist in rates of antibiotic prescribing, with White patients more likely to receive an antibiotic prescription.^23–25^ In this study, White patients were nearly twice as likely to receive an inappropriate antibiotic prescription during the baseline period compared with Black patients. Prescribing differences between races were reduced but persisted during the intervention period. Although this represents a minoritized group receiving more appropriate care, these differences in care by patient race may be driven by implicit bias. Further studies are needed to determine the role of other factors, including socioeconomic status, in receiving inappropriate antibiotic prescriptions.

Reductions in inappropriate antibiotic prescribing were largely seen among clinicians actively engaged in the study, but not among non-actively engaged clinicians. Although some of the interventions were intended to be disseminated from actively engaged clinicians to all clinicians at the center, it is possible that some of the more individual clinician-focused interventions (eg, webinar participation) have a larger impact on reductions in inappropriate prescribing. Alternatively, clinicians who were not direct participants may be joining the UCC during the course of the study, as evidenced by the increasing proportion of non-actively engaged clinicians over time; new clinicians may have less opportunity for interaction with the stewardship intervention content. Clinician turnover at UCCs may be higher compared with many other clinical settings, highlighting the importance of regular engagement in stewardship interventions. As differences in intervention effectiveness may be present between providers, future studies should collect provider-level information including training, years of experience, and number of shifts in order to assess correlations between intervention effectiveness and provider-level factors.

## Limitations

Because data were measured per encounter, it is not known if repeat patients are present in the dataset. Race and ethnicity were collected as one variable, limiting the interpretation of this variable. Conclusions regarding race and ethnicity should be explored in future studies. The proportion of uninsured patients and patients under 1 year or >60 years was relatively low, limiting generalizability. This study did not include a sustainability period to measure if the observed association was sustainable past the intervention period, so we cannot make conclusions regarding long-term effectiveness. Similarly, the brevity of the baseline period along with non-overlapping seasonality between the baseline and intervention periods may affect the reliability of results. However, given that the outcome measure is a rate, the measure and conclusions are unlikely to be changed by fluctuations in the absolute number of diagnoses. Baseline prescribing rates for participants differed from non-participants for bronchitis. This suggests the potential for selection bias, as clinicians who opted to participate in the intervention might have been more inclined towards appropriate antibiotic prescribing practices; however, this difference in baseline prescribing rates was not seen for viral URTI diagnoses (*P* = 0.93). For the intervention, UCCs were encouraged to choose from a package of validated materials best suited to their environment. Although we did not monitor which specific materials were applied at each UCC, precluding evaluation of specific materials, the magnitude of the relative reductions suggests that having UCCs implement materials from a set of validated, publicly available options is an effective approach for reducing inappropriate prescribing overall. This is an intentionally generalizable approach that may be broadly applicable across heterogeneous environments; this may be evidenced by relatively consistent decreases in inappropriate antibiotic prescribing across most sites (Supplemental Figure 4). There was high clinician turnover at participating UCCs, which resulted in a loss of continuity for monthly chart extraction at some sites. Despite this, most UCCs met the data collection targets. Finally, by focusing solely on bronchitis and viral URTIs (tier 3 diagnoses for which antibiotics are never indicated), we could not evaluate the possibility of diagnosis shifting, which may show an increase in other respiratory tract infectious diagnoses (ie, tier 2 diagnoses such as sinusitis or otitis media for which antibiotics are sometimes indicated).^2^ Future studies should expand evaluations to include sinusitis, otitis media, and pharyngitis diagnoses, which are common respiratory diagnoses for inappropriate antibiotic prescribing.

## Conclusion

Overall, the antibiotic stewardship intervention was associated with reduced rates of inappropriate prescribing for both bronchitis and viral URTI diagnoses in this large, geographically diverse collection of UCCs. This study highlights the importance of direct clinician engagement with stewardship efforts and provides a broadly applicable approach to antibiotic stewardship implementation in UCCs.

## Supporting information

Park et al. supplementary material Park et al. supplementary material

## Supplementary material

The supplementary material for this article can be found at https://doi.org/10.1017/ice.2024.213.
