# Supplementary material for: Evaluating an urgent care antibiotic stewardship intervention: a multi-network collaborative effort
Source: Infect Control Hosp Epidemiol. 2025 Jan 8;46(3):266–71. doi: 10.1017/ice.2024.213 (PMC11883647; doi:10.1017/ice.2024.213)
Supplement: Park et al. supplementary material [file S0899823X24002137sup001.docx]

**Supplemental files for “Evaluating the Effect of an Urgent Care Antibiotic Stewardship Intervention: A Multi-Network Collaborative Effort”**

Contents

[Supplemental Figure 1. Statistical Process Control (SPC) charts for inappropriate antibiotic prescribing among bronchitis and viral illness diagnoses 2](#_Toc179547003)

[Supplemental Figure 2. Number of Participating Providers Per Site. 3](#_Toc179547004)

[Supplemental Figure 3. Inappropriate antibiotic prescribing by diagnosis 4](#_Toc179547005)

[Supplemental Figure 4. Inappropriate antibiotic prescribing rates by month across 25 UCCs over the study period. 5](#_Toc179547006)

[Supplemental Table 1. Revised Standards for Quality Improvement Reporting Excellence (SQUIRE 2.0) Checklist 6](#_Toc179547007)

[Supplemental Table 2: ICD-10 codes used for chart extraction. 9](#_Toc179547008)

[Supplemental Table 3. Concurrent diagnoses leading to chart exclusion from inappropriate antibiotic prescribing. 9](#_Toc179547009)

[Supplemental Table 4. Intervention Choices to Implement During the Second and Third PDSA Cycles of the Study. 10](#_Toc179547010)

[Supplemental Table 5. Urgent care center encounter patient characteristics by clinician participation status. 11](#_Toc179547011)

[Supplemental Table 6. 12](#_Toc179547012)

# Supplemental Figure 1. Statistical Process Control (SPC) charts for inappropriate antibiotic prescribing among bronchitis and viral illness diagnoses

**
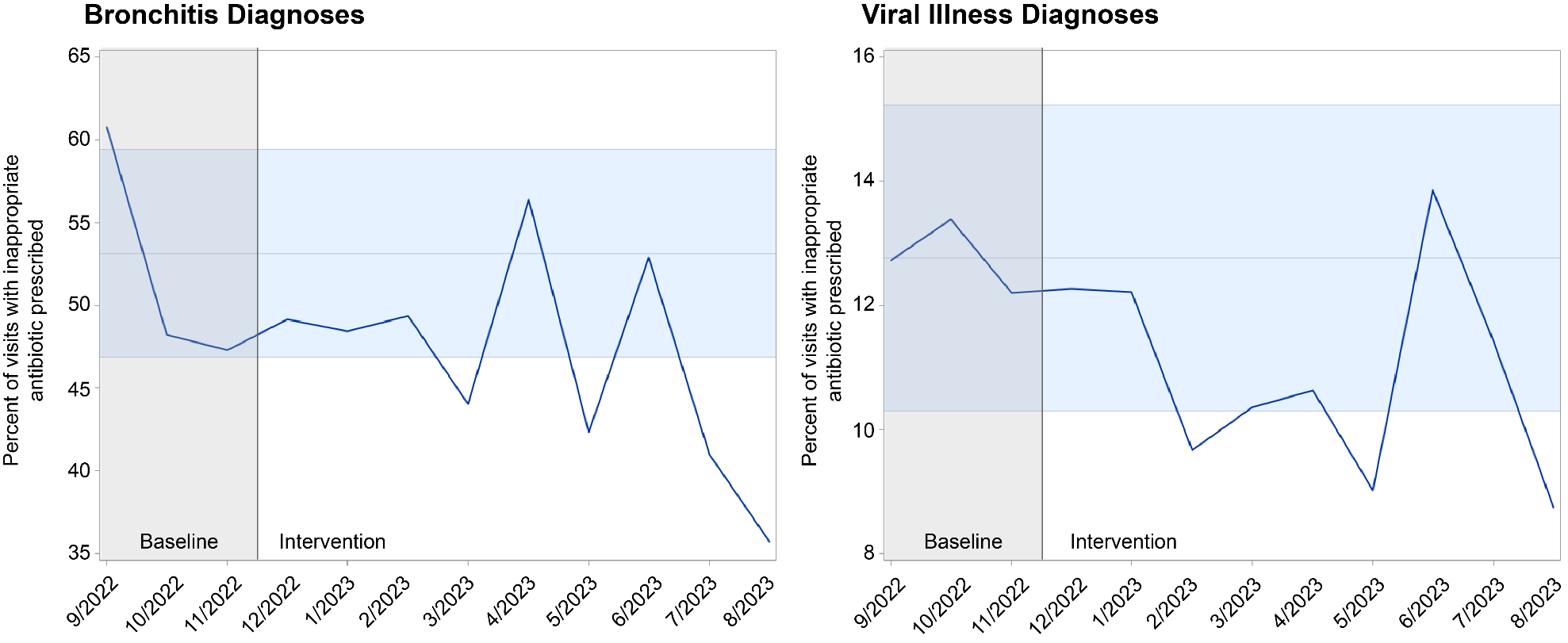
**

SPC chart upper and lower control limits derived using 2 standard deviations from the mean baseline (Sep to Nov 2022) inappropriate prescribing rates, and displayed as shaded regions in the figure. For bronchitis, the lower control limit is 46.9% and the upper control limit is 59.4%. For viral illness, the lower control limit is 10.3% and the upper control limit is 15.2%.

# Supplemental Figure 2. Number of Participating Providers Per Site.


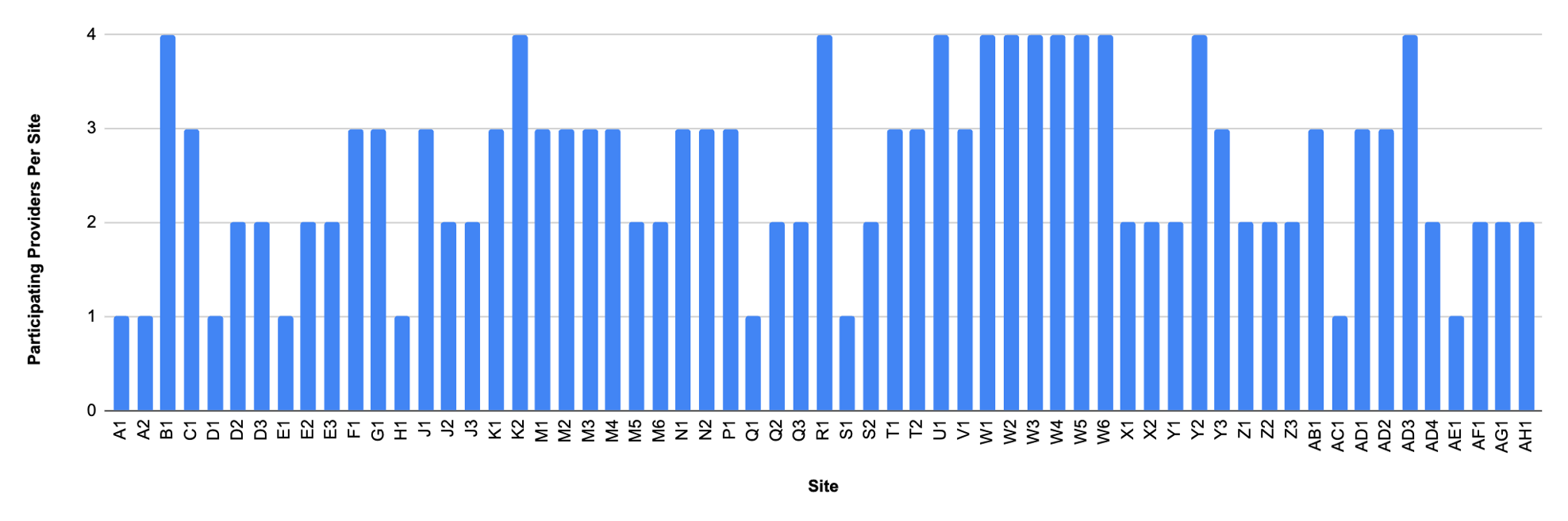


# Supplemental Figure 3. Inappropriate antibiotic prescribing by diagnosis


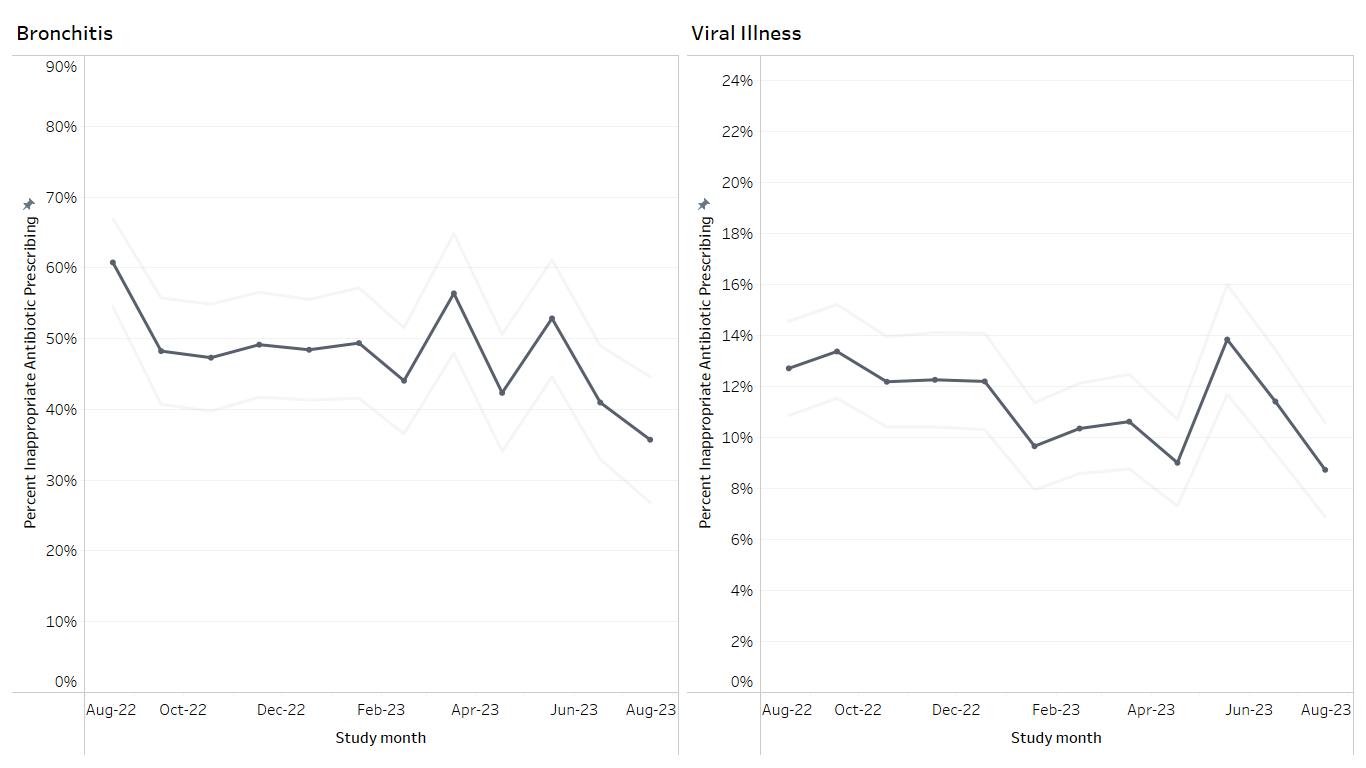


Percent of urgent care encounters with an inappropriate antibiotic prescription by month, diagnosis. Faded lines represent the 95% confidence intervals for the inappropriate antibiotic prescribing rate.

# Supplemental Figure 4. Inappropriate antibiotic prescribing rates by month across 25 UCCs over the study period.


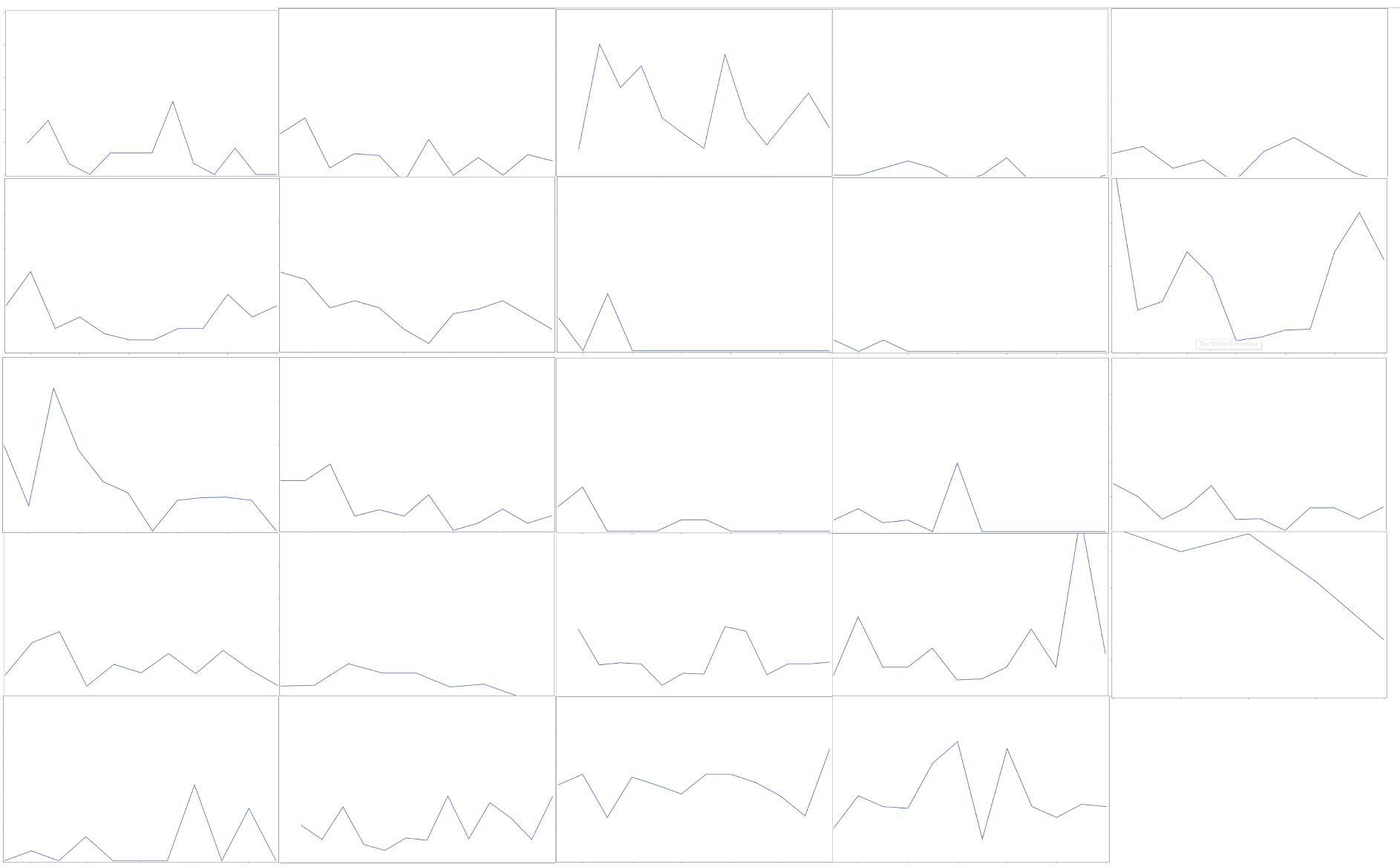


Panel displays the inappropriate antibiotic prescribing rates by calendar month beginning with the baseline period (3 months) followed by the intervention period (9 months). UCCs with at least 35 baseline charts entered are displayed.

# Supplemental Table 1. Revised Standards for Quality Improvement Reporting Excellence (SQUIRE 2.0) Checklist

|  |  | Reporting Item | Page Number |
| --- | --- | --- | --- |
| **Title** |  |  |  |
|  | [#1](https://www.goodreports.org/reporting-checklists/squire/info/#1) | Indicate that the manuscript concerns an initiative to improve healthcare (broadly defined to include the quality, safety, effectiveness, patientcenteredness, timeliness, cost, efficiency, and equity of healthcare) | 1 |
| **Abstract** |  |  |  |
|  | [#02a](https://www.goodreports.org/reporting-checklists/squire/info/#02a) | Provide adequate information to aid in searching and indexing | 2-3 |
|  | [#02b](https://www.goodreports.org/reporting-checklists/squire/info/#02b) | Summarize all key information from various sections of the text using the abstract format of the intended publication or a structured summary such as: background, local problem, methods, interventions, results, conclusions | 2-3 |
| **Introduction** |  |  |  |
| Problem description | [#3](https://www.goodreports.org/reporting-checklists/squire/info/#3) | Nature and significance of the local problem | 4 |
| Available knowledge | [#4](https://www.goodreports.org/reporting-checklists/squire/info/#4) | Summary of what is currently known about the problem, including relevant previous studies | 4 |
| Rationale | [#5](https://www.goodreports.org/reporting-checklists/squire/info/#5) | Informal or formal frameworks, models, concepts, and / or theories used to explain the problem, any reasons or assumptions that were used to develop the intervention(s), and reasons why the intervention(s) was expected to work | 4 |
| Specific aims | [#6](https://www.goodreports.org/reporting-checklists/squire/info/#6) | Purpose of the project and of this report | 4 |
| **Methods** |  |  |  |
| Context | [#7](https://www.goodreports.org/reporting-checklists/squire/info/#7) | Contextual elements considered important at the outset of introducing the intervention(s) | 5-8 |
| Intervention(s) | [#08a](https://www.goodreports.org/reporting-checklists/squire/info/#08a) | Description of the intervention(s) in sufficient detail that others could reproduce it | 5-8 |
| Intervention(s) | [#08b](https://www.goodreports.org/reporting-checklists/squire/info/#08b) | Specifics of the team involved in the work | 5-8 |
| Study of the Intervention(s) | [#09a](https://www.goodreports.org/reporting-checklists/squire/info/#09a) | Approach chosen for assessing the impact of the intervention(s) | 5-8 |
| Study of the Intervention(s) | [#09b](https://www.goodreports.org/reporting-checklists/squire/info/#09b) | Approach used to establish whether the observed outcomes were due to the intervention(s) | 5-8 |
| Measures | [#10a](https://www.goodreports.org/reporting-checklists/squire/info/#10a) | Measures chosen for studying processes and outcomes of the intervention(s), including rationale for choosing them, their operational definitions, and their validity and reliability | 5-8 |
| Measures | [#10b](https://www.goodreports.org/reporting-checklists/squire/info/#10b) | Description of the approach to the ongoing assessment of contextual elements that contributed to the success, failure, efficiency, and cost | 5-8 |
| Measures | [#10c](https://www.goodreports.org/reporting-checklists/squire/info/#10c) | Methods employed for assessing completeness and accuracy of data | 5-8 |
| Analysis | [#11a](https://www.goodreports.org/reporting-checklists/squire/info/#11a) | Qualitative and quantitative methods used to draw inferences from the data | 7-8 |
| Analysis | [#11b](https://www.goodreports.org/reporting-checklists/squire/info/#11b) | Methods for understanding variation within the data, including the effects of time as a variable | 7-8 |
| Ethical considerations | [#12](https://www.goodreports.org/reporting-checklists/squire/info/#12) | Ethical aspects of implementing and studying the intervention(s) and how they were addressed, including, but not limited to, formal ethics review and potential conflict(s) of interest | 5 |
| **Results** |  |  |  |
|  | [#13a](https://www.goodreports.org/reporting-checklists/squire/info/#13a) | Initial steps of the intervention(s) and their evolution over time (e.g., time-line diagram, flow chart, or table), including modifications made to the intervention during the project | 8-9 |
|  | [#13b](https://www.goodreports.org/reporting-checklists/squire/info/#13b) | Details of the process measures and outcome | 8-10 |
|  | [#13c](https://www.goodreports.org/reporting-checklists/squire/info/#13c) | Contextual elements that interacted with the intervention(s) | 8-9 |
|  | [#13d](https://www.goodreports.org/reporting-checklists/squire/info/#13d) | Observed associations between outcomes, interventions, and relevant contextual elements | 8-10 |
|  | [#13e](https://www.goodreports.org/reporting-checklists/squire/info/#13e) | Unintended consequences such as unexpected benefits, problems, failures, or costs associated with the intervention(s). | 8-10 |
|  | [#13f](https://www.goodreports.org/reporting-checklists/squire/info/#13f) | Details about missing data | 8-10 |
| **Discussion** |  |  |  |
| Summary | [#14a](https://www.goodreports.org/reporting-checklists/squire/info/#14a) | Key findings, including relevance to the rationale and specific aims | 10-11 |
| Summary | [#14b](https://www.goodreports.org/reporting-checklists/squire/info/#14b) | Particular strengths of the project | 10-11 |
| Interpretation | [#15a](https://www.goodreports.org/reporting-checklists/squire/info/#15a) | Nature of the association between the intervention(s) and the outcomes | 10-11 |
| Interpretation | [#15b](https://www.goodreports.org/reporting-checklists/squire/info/#15b) | Comparison of results with findings from other publications | 10-11 |
| Interpretation | [#15c](https://www.goodreports.org/reporting-checklists/squire/info/#15c) | Impact of the project on people and systems | 10-11 |
| Interpretation | [#15d](https://www.goodreports.org/reporting-checklists/squire/info/#15d) | Reasons for any differences between observed and anticipated outcomes, including the influence of context | 10-11 |
| Interpretation | [#15e](https://www.goodreports.org/reporting-checklists/squire/info/#15e) | Costs and strategic trade-offs, including opportunity costs | 10-11 |
| Limitations | [#16a](https://www.goodreports.org/reporting-checklists/squire/info/#16a) | Limits to the generalizability of the work | 12 |
| Limitations | [#16b](https://www.goodreports.org/reporting-checklists/squire/info/#16b) | Factors that might have limited internal validity such as confounding, bias, or imprecision in the design, methods, measurement, or analysis | 12 |
| Limitations | [#16c](https://www.goodreports.org/reporting-checklists/squire/info/#16c) | Efforts made to minimize and adjust for limitations | 12 |
| Conclusion | [#17a](https://www.goodreports.org/reporting-checklists/squire/info/#17a) | Usefulness of the work | 12-13 |
| Conclusion | [#17b](https://www.goodreports.org/reporting-checklists/squire/info/#17b) | Sustainability | 12-13 |
| Conclusion | [#17c](https://www.goodreports.org/reporting-checklists/squire/info/#17c) | Potential for spread to other contexts | 12-13 |
| Conclusion | [#17d](https://www.goodreports.org/reporting-checklists/squire/info/#17d) | Implications for practice and for further study in the field | 12-13 |
| Conclusion | [#17e](https://www.goodreports.org/reporting-checklists/squire/info/#17e) | Suggested next steps | 12-13 |
| **Other information** |  |  |  |
| Funding | [#18](https://www.goodreports.org/reporting-checklists/squire/info/#18) | Sources of funding that supported this work. Role, if any, of the funding organization in the design, implementation, interpretation, and reporting | 13 |

The SQUIRE 2.0 checklist is distributed under the terms of the Creative Commons Attribution License CC BY-NC 4.0. This checklist was completed on 12. April 2024 using <https://www.goodreports.org/>, a tool made by the [EQUATOR Network](https://www.equator-network.org) in collaboration with [Penelope.ai](https://www.penelope.ai)

# Supplemental Table 2: ICD-10 codes used for chart extraction.

ICD-10 codes used for data extraction.

| Condition | ICD-10 codes |
| --- | --- |
| Bronchitis | J20.0, J20.1, J20.2, J20.3, J20.4, J20.5, J20.6, J20.7, J20.8, J20.9, J21.0, J21.1, J21.8, J21.9, J40, J41.0, J41.1, J41.8, U07.1 |
| Viral upper respiratory tract infections | J00, J01.0, J01.1, J01.2, J01.3, J01.4, J01.8, J01.9, J02.0, J02.8, J02.9, J03.8, J03.9, J04.0, J04.1, J04.2, J04.3, J05.0, J05.1, J06.0, J06.9, H65.0, H65.1, H66.0, H66.4 |

# Supplemental Table 3. Concurrent diagnoses leading to chart exclusion from inappropriate antibiotic prescribing.

| Conditions | ICD-10 Codes |
| --- | --- |
| ​​Acute maxillary sinusitis, frontal sinusitis, ethmoidal sinusitis, sphenoidal sinusitis, and pansinusitis, suppurative acute otitis media, strep pharyngitis, central venous catheter infections, infection after immunization, bites and scratches, infections due to specific pathogens (e.g., Salmonella), sexually transmitted diseases, neutropenia, cellulitis, abscess, otitis externa, chronic otitis media, pneumonia, chronic sinusitis, impetigo, lymphangitis, lymphadenitis, acne and other skin conditions, septic arthritis, osteomyelitis and discitis, myositis, pyelonephritis, cystitis, urinary tract infection, salpingitis, peritonitis, pelvic infections, sepsis, and bacteremia | J01.0n, J01.1n, J01.2n, J01.3n, J01.4n,  J01.8n, J01.9n, H66.4, H66.4n, H66.00, H66.00n, J02.0, J03.0, T80.2n, T88.0n, W50.3n, W53.n1Xn, W54.0XXn, W55.n1Xn, W56.n1Xn, W58.n1Xn, W59.n1Xn, W61.n1xn, Y04.1xxn, A01.0n, A01.n, A02.n, A03.n, A04.n, A18.0n, A22.7, A23.n, A26-27.n, A28.0, A28.2-9, A32.n, A33, A37.n, A38.1-9, A40-41.n, A42.7, A48-59.n, A63.8, A64, A69.2n, A74.8-9n, B37.7, B78.1, B95.n, B96.n, D70.n, H00.036, H05.01n, H60.0-3n, H62.4n, H66.1-3n, I00, I01.n, J13-8.n, J20.0-2, J32.n, J36, J39.0-1, K04.0, K04.4, K04.6-7, K12.2, K61.n, L01-8.n except L03.213 and L08.1, L70.n, L73.0, L88, L92.8, L98.0, L98.3, M00-1.n, M46.2-3n, M60.0n,  M86.n, N11.0-1, 13N.6, N15.1, N15.9, N16, N30.n, N34.0-2, N39.0, N70.n, N71-7.n, N98.0, P36-9.n, R65.n, and/ or R78.81 |
| 1. An “n” within or at the end of a code (eg, K61.n) means that any ICD-10 code with any number or letter in that position is included. | |

# Supplemental Table 4. Intervention Choices to Implement During the Second and Third PDSA Cycles of the Study.

| **Intervention Category** | **Specific Intervention^1^** |
| --- | --- |
| Patient Education | ABS Trifold Handout*  ABS Two Page Handout  Bronchitis Two Page Handout  Improving Antibiotics Two Page Handout*  Runny Nose One Page Handout  Virus or Bacteria One Page Poster* |
| Patient Engagement | Consumer Report When to Say No to Antibiotics for Infections  How to Say no To Antibiotics  CDC Letter Daycare  CDC Patient Experience (Video)  New York State Department of Health: Educating Patients About Antibiotic Usage (Video)  RX for Viral Illness*  CDC Rx Relief for Viral Illness* |
| Clinician Education | Dialogue Around Respiratory Illness Treatment (DART) Program  Antibiotic Resistance Report 2019  When Antibiotic Experts Say No to Antibiotics  CDC Core Elements of Outpatient Antibiotic Stewardship Checklist  CDC Core Elements of Antibiotic Stewardship Report  How to Prescribe Fewer Unnecessary Antibiotics: Patient Talking Points  CDC Antibiotic Stewardship Training Series |
| Treatment Guidelines | Adult Outpatient Recommendations  Pediatric Outpatient Recommendations |
| Signage | CDC 8 Ways to Be Antibiotics Aware Poster  “Do Antibiotics Have Side Effects?” Infographic*  “Do I need Antibiotics?” Infographic*  Improve Outpatient Antibiotic Use Sign  PSA Right Tool* |
| Social | Antibiotics Resistance by the Numbers  Do I Really Need Antibiotics?  What’s the Best Treatment Plan for Me Today?  Virus or Bacteria? |
|  |  |

^1^From https://urgentcareassociation.org/about/strategic-initiatives/abs-change-packet/

*Material offered in both English and Spanish

# Supplemental Table 5. Urgent care center encounter patient characteristics by clinician participation status.

| **Characteristic** | **Baseline Period** | | **Intervention Period** | |
| --- | --- | --- | --- | --- |
|  | Actively engaged | Non-actively engaged | Actively engaged | Non-actively engaged |
| Total visits (row %) | 2100 (47.6) | 2310 (52.4) | 5431 (49.8) | 5476 (50.2) |
| **Race/Ethnicity** |  |  |  |  |
| Black | 253 (12.1) | 269 (11.7) | 494 (9.1) | 555 (10.1) |
| White | 1312 (62.5) | 1391 (60.2) | 3712 (68.4) | 3344 (61.1) |
| Hispanic | 153 (7.3) | 182 (7.9) | 506 (9.3) | 463 (8.5) |
| Other | 382 (18.2) | 468 (20.3) | 719 (13.2) | 1114 (20.3) |
| **Insurance** |  |  |  |  |
| Commercial | 1363 (64.9) | 1383 (59.9) | 3620 (66.7) | 3102 (56.7) |
| Military | 35 (1.7) | 22 (1.0) | 97 (1.8) | 64 (1.2) |
| Public | 554 (26.4) | 676 (29.3) | 1506 (27.7) | 1706 (31.2) |
| None | 67 (3.2) | 89 (3.9) | 134 (2.5) | 221 (4.0) |
| Unsure | 81 (3.9) | 140 (6.1) | 74 (1.4) | 382 (7.0) |
| **Age** |  |  |  |  |
| 0-11m | 57 (2.7) | 43 (1.9) | 171 (3.2) | 115 (2.1) |
| 1-20y | 1146 (54.6) | 1003 (43.4) | 2,982 (54.9) | 1976 (36.1) |
| 21-40y | 402 (19.1) | 618 (26.8) | 1,090 (20.1) | 1662 (30.3) |
| 41-60y | 278 (13.2) | 396 (17.1) | 643 (11.8) | 1016 (18.5) |
| 61-80y | 194 (9.2) | 224 (9.7) | 489 (9.0) | 635 (11.6) |
| 81 and over | 23 (1.1) | 26 (1.1) | 56 (1.0) | 72 (1.3) |

Engagement status missing from a total of n=68 charts.

Percentages are column percentages unless otherwise noted.

# Supplemental Table 6. Inappropriate antibiotic prescribing by study time period and clinician participation status.

|  | **Inappropriate antibiotic prescribed / number of encounters (%)** | | | | **p-value*** | | |
| --- | --- | --- | --- | --- | --- | --- | --- |
|  | 1. **Baseline** | 1. **Intervention Month 1-2** | 1. **Intervention Month 3-7** | 1. **Intervention Month 8-9** | A v B | A v C | A v D |
| **Bronchitis or Viral URTI** | 799/4439  (18.0) | 466/2711 (17.2) | 933/6141 (15.2) | 284/2094 (13.6) | 0.384 | **<0.001** | **<0.001** |
| Actively engaged clinician | 333/2100  (15.9) | 171/1440 (11.9) | 262/3019 (8.7) | 80/972 (8.2) | **<0.001** | **<0.001** | **<0.001** |
| Non-actively engaged clinician | 450/2310  (19.5) | 293/1262 (23.2) | 670/3092 (21.7) | 204/1122 (18.2) | **0.009** | 0.0497 | 0.364 |
| **Bronchitis Diagnoses Overall** | 305/573  (53.2) | 177/363 (48.8) | 357/734 (48.6) | 99/255 (38.8) | 0.183 | **0.010** | **<0.001** |
| Actively engaged clinician | 92/230  (40.0) | 48/135 (35.6) | 65/234 (27.8) | 16/80 (20.0) | 0.399 | **0.005** | **0.001** |
| Non-actively engaged clinician | 201/329  (61.1) | 128/227 (56.4) | 291/487 (59.8) | 83/175 (47.4) | 0.268 | 0.701 | **0.003** |
| **Viral URTI Diagnoses Overall** | 494/3866  (12.8) | 289/2348 (12.3) | 576/5407 (10.7) | 185/1839 (10.1) | 0.589 | **0.002** | **0.003** |
| Actively engaged clinician | 241/1870  (12.9) | 123/1305 (9.4) | 197/2785 (7.1) | 64/892 (7.2) | **0.003** | **<0.001** | **<0.001** |
| Non-actively engaged clinician | 249/1981  (12.6) | 165/1035 (15.9) | 379/2605 (14.5) | 121/947 (12.8) | **0.011** | 0.053 | 0.874 |

*Uncorrected chi-square p-value from two-tailed test
